# Risk stratification and multidisciplinary management strategies for pregnancy complicated by pulmonary arterial hypertension: a narrative review

**DOI:** 10.3389/fendo.2026.1911305

**Published:** 2026-08-10

**Authors:** Minghui Mao, Feng Huang, Maochun Yang, Lanfang Chen, Yan Liu

**Affiliations:** Zhongjiang Maternal and Child Health-Care Hospital, Deyang, Sichuan, China

**Keywords:** health risks, management strategy, MDT, pregnancy, pulmonary arterial hypertension

## Abstract

Pulmonary arterial hypertension (PAH) complicating pregnancy is a severe condition that poses a significant threat to maternal and foetal health. Despite recent improvements in outcomes for pregnant women with PAH, attributable to continuous updates in diagnostic and treatment strategies and the implementation of multidisciplinary team management, further research is needed on the clinical applicability of risk stratification systems and standardised processes for multidisciplinary management. This article provides a narrative review of the potential impact of pregnancies complicated by PAH on mothers and foetuses, as well as management strategies during pregnancy and childbirth. The aim is to summarise the health risks and management strategies for pregnant women in the preconception, antenatal, and postpartum periods, thereby providing a reference for clinical practice.

## 1 Introduction

Pulmonary arterial hypertension (PAH) is a progressive pathophysiological syndrome characterised by pulmonary vascular endothelial injury, vascular remodelling, and a progressive elevation of pulmonary vascular resistance (1). Right heart catheterisation at rest is the gold standard for diagnosis, with a definitive threshold of mean pulmonary artery pressure ≥20 mmHg (2). PAH demonstrates a striking female predominance, with an incidence roughly fourfold higher in women and peak prevalence occurring during reproductive years (3). Pregnancies complicated by PAH confer elevated risks of life-threatening complications, including pulmonary edema, peripartum right heart failure, and venous thromboembolism (4), with maternal mortality rates reaching as high as 12% (5). In recent years, ongoing advances in PAH diagnostic and therapeutic modalities, coupled with refined perinatal care protocols, have driven a consistent decline in maternal mortality among PAH-affected pregnant individuals. These observations underscore that standardised, tailored perinatal care for PAH remains central to optimising overall perinatal outcomes (6).

According to the modified World Health Organisation (mWHO) pregnancy risk classification, patients with PAH arising from any etiology are stratified into the highest risk level (Level IV) (7). The Chinese ‘Expert Consensus on the Diagnosis and Treatment of Pregnancy Complicated with Heart Disease (2016)’ clearly states that pregnant women assigned to risk Level V (including those with PAH) should undergo pregnancy termination and implement reliable contraception (8). The mWHO system is designed specifically to assess the overall maternal cardiovascular risk associated with pregnancy and to guide the decision on whether to proceed with or terminate pregnancy. In contrast, the ESC/ERS three-tier PAH scale (low/intermediate/high risk) is a prognostic tool developed primarily for non-pregnant PAH patients, intended to guide PAH-targeted therapy and predict long-term outcomes (7). These two systems serve fundamentally different purposes and should not be used interchangeably (1). Importantly, the haemodynamic thresholds and prognostic performance of the ESC/ERS scale remain unvalidated in pregnant patients, posing a substantial clinical challenge. Furthermore, 56% of such patients currently opt to continue their pregnancies (9), posing a substantial challenge to precise, risk-stratified clinical management (10).

Currently, the multidisciplinary team (MDT) approach to managing pregnancy-associated PAH relies predominantly on empirical collaborative practices. No unified, standardised, risk-stratified clinical management framework has been widely established (11). Several recent reviews have addressed general management principles for PAH in pregnancy, but they often treat risk stratification superficially, without systematically comparing the applicability of different risk tools in the gestational context (12, 13), nor do they provide a structured MDT workflow aligned with dynamic risk changes throughout the perinatal period (1, 14). Moreover, the specific outcomes and management nuances across diverse PAH aetiologies are frequently aggregated, obscuring important prognostic differences (15). To address these gaps, this review summarises the etiological classification, risk stratification criteria, core management strategies, and multidisciplinary collaboration workflows for pregnancy-associated PAH. Specifically, we (i) clarify the distinct purposes and limitations of the mWHO and ESC/ERS risk systems in pregnancy; (ii) present aetiology-specific maternal and foetal outcome data to guide tailored counselling; (iii) propose a dynamic, risk-stratified monitoring protocol across antenatal, intrapartum, and postpartum phases; and (iv) outline a practical MDT operational framework that integrates clinical warning signs and escalation pathways. By doing so, we aim to identify critical gaps in current practice and provide actionable references for standardised diagnosis and treatment, thereby offering a novel contribution beyond existing narrative reviews.

## 2 Pathogenesis and maternal-foetal risks of pregnancy complicated by PAH

### 2.1 Pathophysiological mechanisms

During pregnancy, elevated blood volume and cardiac output, alongside progesterone-mediated pulmonary vasodilation, should augment pulmonary blood flow to satisfy metabolic demands. However, patients with PAH, exhibit pulmonary arteriolar wall thickening and vascular remodelling, which impairs adaptive vasodilation. This triggers progressive elevation of pulmonary vascular resistance (PVR) and a sharp marked surge in right ventricular afterload, ultimately precipitating right heart failure (2, 5, 16). The risk peaks notably between gestational weeks 32 to 34, during labour, and within the first three days postpartum (17, 18).

Patients with PAH have been shown to exhibit pulmonary vascular endothelial dysfunction, platelet activation, and abnormalities in the coagulation cascade. These factors render patients susceptible to the development of pulmonary arteriolar microthrombi. The hypercoagulable state of the blood during pregnancy further increases the risk of thromboembolism, posing a particularly severe risk to patients with chronic thromboembolic pulmonary hypertension (CTEPH) (19, 20). Several factors associated with childbirth, including but not limited to blood loss, fluid transfer, severe pain, and metabolic disorders, have been demonstrated to exacerbate pulmonary vascular resistance and increase the thromboembolism risk (21). Notably, patients with Eisenmenger syndrome are susceptible to coagulation dysfunction, which may coexist with an increased bleeding risk attributable to hepatic dysfunction and thrombocytopenia (22).

### 2.2 Clinical applications of genetic testing in pregnancy complicated by PAH

Genetic testing has evolved from a supportive diagnostic tool to a central component of clinical decision-making in the pregnancy management of pulmonary arterial hypertension (PAH). Germline mutations, particularly in BMPR2—found in over 70% of hereditary PAH and approximately 20% of idiopathic PAH cases—are central to PAH pathogenesis (23–26). Current consensus recommends that genetic counselling and testing be offered to patients with idiopathic PAH, familial PAH, pulmonary veno-occlusive disease/pulmonary capillary haemangiomatosis, anorexigen-induced PAH, and congenital heart disease-associated PAH (27). A PAH gene panel sequencing approach should include at least the 12 core genes with “strong” or “definitive” evidence for gene-disease association-ACVRL1, ATP13A3, BMPR2, CAV1, EIF2AK4, ENG, GDF2, KCNK3, KDR, SMAD9, SOX17, and TBX4-with supplementary methods to detect copy number variations, particularly for BMPR2, ACVRL1, ENG, and TBX4 (27).

In the context of pregnancy, the clinical utility of genetic testing extends across three domains: preconception genetic counselling, cascade family screening, and reproductive planning. Women known to carry pathogenic gene mutations should receive professional genetic counselling before pregnancy to assess their own risk of developing or worsening PAH during gestation, and the risk of transmitting the mutation to their offspring. Genetic counselling should address the autosomal dominant inheritance with incomplete penetrance that characterises most PAH-associated variants—for example, BMPR2 mutations exhibit a penetrance of approximately 42% in females and 14% in males (28) —and should also cover the potential psychosocial impact on reproductive decisions, insurance, and psychological well-being (7). Once a pathogenic variant is identified in a proband, cascade testing should be offered to first-degree relatives (including sisters and daughters) if they are contemplating pregnancy, and asymptomatic carriers should undergo regular clinical surveillance to enable early diagnosis and treatment initiation (27). For couples at risk of transmitting PAH-associated mutations, preimplantation genetic testing for monogenic disorders (PGT-M) allows the selection of embryos free of disease-causing mutations (29). When combined with *in vitro* fertilisation (IVF) and gestational surrogacy, this approach enables biological parenthood while completely avoiding the cardiopulmonary risks of pregnancy in women with PAH (30). For those who decline surrogacy for personal, cultural, or legal reasons and choose to carry a pregnancy themselves, a conservative strategy using natural cycle frozen embryo transfer with single euploid embryos can reduce—but not eliminate—risk (30). Collectively, these applications underscore that the clinical relevance of genetic testing in pregnancy lies not merely in molecular characterisation, but in its capacity to inform actionable reproductive decisions, guide family screening, and support multidisciplinary preconception and antenatal care (27, 30).

### 2.3 Pregnancy outcomes with different aetiologies

The risk of adverse pregnancy outcomes varies depending on the underlying cause of PAH (see **Table 1**). The risk of major adverse cardiovascular events (MACE), including heart failure and arrhythmia, is significantly elevated in pregnant women with PAH compared to pregnant women without PAH, with a risk ratio of 62. In patients diagnosed with PAH associated with congenital heart disease, the incidence of MACE has been documented as 15.3%. However, in patients diagnosed with PAH associated with cardiomyopathy and valvular heart disease, the prevalence increases to 64.1%. This finding suggests that patients with PAH associated with left heart disease may have a more poorer prognosis (45). Long-term follow-up results demonstrate that the post-discharge five-year cumulative survival rate for pregnant women diagnosed with severe PAH (pulmonary artery systolic pressure (PASP) ≥50 mmHg) is only 35%. In contrast, it can reach 90% in those with moderate PAH (30 ≤ PASP ≤ 49 mmHg) (46). This finding suggests that pulmonary artery pressure levels may be a significant factor in determining long-term prognosis.

**Table 1:** Maternal and foetal/neonatal outcomes by PAH etiology.

PAH subtype	Maternal outcomes	Fetal/neonatal outcomes	Key management notes	Source of evidence: references
CHD-related PAH (Eisenmenger)	Mortality: 17–30% (higher in high-risk cohorts) Morbidity: RHF, arrhythmia, hypoxemic crisis, postpartum hemodynamic collapse	Preterm birth >60% (up to 80%); low birth weight; high perinatal mortality	Highest risk in Eisenmenger; avoid pregnancy; if continued, MDT in expert centre	Observational study: (31–35)
CTD-PAH (SSc, SLE)	Mortality: ~10–15% (depends on disease activity and cardiac involvement) Morbidity: RHF, PAH crisis, cardiac arrest	Preterm birth common; FGR; neonatal survival better than Eisenmenger	Manage with rheumatology & cardiology; immunosuppressants; outcomes better if low SLE activity	Observational study: (36) Review: (37–39)
IPAH/Heritable PAH	Mortality: 10–20% (modern cohorts) Morbidity: RHF (mainly postpartum), thromboembolic events, need for early C-section	Preterm birth 50–70%; low birth weight; live birth rate 80–90% if well-controlled	Better outcomes if on targeted therapy pre-pregnancy and low-risk status	Observational study: (40–42) Review: (5, 43)
Other/mixed etiologies (portal HTN, HIV, Graves’, HHT)	Mortality: ~8%; higher with severe PH (OR ~5.6) Morbidity: RHF, liver dysfunction, thrombocytopenia, postpartum haemorrhage	Preterm birth 20–27%; low birth weight 19–22%; NICU admission ~16%	MDT core; manage underlying disease; postpartum ICU ≥72 h	Observational study: (44) 44 Review: (10, 11, 31)

It is important to interpret these findings with appropriate caution. The available evidence is derived predominantly from retrospective cohort studies and single-centre case series, which are subject to considerable heterogeneity in patient selection, management protocols, and PAH-targeted therapy availability across different eras and regions. Such methodological limitations may affect the generalisability of the reported outcome estimates. Nevertheless, the consistent pattern of worse outcomes in left heart disease-associated PAH and in patients with higher PASP levels provides a useful evidence base for risk stratification. From a clinical standpoint, these aetiology-specific differences should inform a tiered approach to care: patients with CHD-PAH, while still at significant risk, may have relatively more favourable outcomes and could be candidates for carefully planned pregnancy under close multidisciplinary supervision; in contrast, those with left heart disease-associated PAH or severe pulmonary hypertension should be counselled more strongly against pregnancy, and if pregnancy proceeds, should be managed in expert centres with the highest level of monitoring and preparedness for rapid intervention. These observations reinforce the need for aetiology-guided, individualised risk assessment and multidisciplinary decision-making in the management of pregnancies complicated by PAH.

## 3 Risk stratification of pregnancies complicated by PAH

### 3.1 Core indicators for risk stratification

The physiological haemodynamic changes during pregnancy can confound traditional risk indicators; consequently, no pregnancy-specific PAH stratification scale is currently available in clinical practice. Therefore, the stratification logic validated in non-pregnant populations is adapted for use in pregnant patients. Core indicators span multiple dimensions, including pulmonary vascular pressure, cardiac functional reserve, neurohormonal activation, and tissue oxygenation as shown in **Table 2** (7, 29). Establishing common stratification thresholds for each indicator facilitates rapid clinical assessment and dynamic monitoring. In practice, however, these thresholds must be interpreted alongside the patient’s underlying aetiology, disease trajectory, and MDT evaluation to ensure that management strategies are not driven by a single parameter. However, it should be noted with caution that the validity and prognostic value of the aforementioned indicators in pregnant populations are subject to various degrees of limitation. The normal reference ranges of BNP/NT-proBNP exhibit physiological fluctuations with advancing gestational age, and are influenced by factors such as physiological anaemia of pregnancy and increased glomerular filtration rate, which limit their clinical utility in cardiovascular risk assessment during pregnancy (50); Plasma volume expansion and increased cardiac output during pregnancy may also mask the true extent of elevation in pulmonary arterial pressure at rest, leading to a potential underestimation of pulmonary artery systolic pressure (PASP) measured by a single echocardiographic examination. Furthermore, studies on the optimal warning threshold of prepartum SpO_2_ in pregnant women with PAH remain limited, and lack external validation in large cohorts (14); Furthermore, the six-minute walk distance, as a core indicator in the ESC/ERS stratification, remains understudied in pregnant populations, with the greatest challenge being the lack of large scale, standardised baseline data for pregnant women (51, 52). Therefore, the current risk stratification for PAH during pregnancy is not a dedicated system validated in pregnant populations. Its threshold accuracy and prognostic relevance require dynamic adjustment in clinical practice by integrating the patient’s underlying aetiology, symptomatic evolution, and multidisciplinary team judgement, so as to avoid management decisions based on a single indicator.

**Table 2:** Risk stratification thresholds and clinical implications for pregnancy with PAH.

Risk indicator	Low	Intermediate	High	Very high	Clinical implication & action	References
PASP (mmHg)	<50	50–69	≥70	+RHF signs	≥70 mmHg → MACE risk ↑21.0-fold → MDT emergency evaluation; if continue pregnancy, expert centre care	(32, 34)
NYHA/WHO-FC	I–II	–	III–IV	IV	III–IV → hospitalization or weekly monitoring; strengthen MDT follow-up	(43, 47)
BNP (ng/L)	<50	50–300	>300	+persistent elevation & clinical symptoms	>300 ng/L → independent predictor of adverse events (OR 17.3) → MDT emergency evaluation, consider pregnancy termination	(7, 29, 48)
NT-proBNP (ng/L)	<300	300–1400	>1400	+persistent elevation & clinical symptoms	>1400 ng/L = ESC/ERS 2022 high-risk threshold; >1520 ng/L = strongest predictor of death (sens 75%, spec 85.6%); urgent intervention if worsening	(7, 29, 49)
SpO_2_	≥95%	90–94%	<90%	+hypoxemic symptoms	<90% → oxygen therapy; independent risk factor for MACE (SpO_2_ >90% protective, OR 0.05); 90–94% borderline; emergency evaluation if symptoms	(49)

### 3.2 Additional evaluation

#### 3.2.1 Echocardiography

This non-invasive tool is used to monitor right ventricular ejection fraction during pregnancy, which is critical for identifying right ventricular (RV) dysfunction in patients with PAH. Tricuspid annular plane systolic excursion (TAPSE) measures the longitudinal component of RV contraction, whereas changes in RV area fraction evaluate both longitudinal and transverse components. Lower TAPSE levels generally indicate impaired RV function. Furthermore, RV tissue Doppler imaging, which measures peak systolic velocity and myocardial function, facilitates early clinical detection of RV dysfunction in gestational PAH. Nevertheless, no established echocardiographic threshold currently exists specifically for pregnancy-associated PAH (53), and further research is warranted.

Right Heart Catheterisation (RHC)

RHC remains the gold standard for diagnosing PAH. Its parameters aid in risk stratification for adverse events or death and guide treatment decisions. However, owing to its invasive nature and the risk of foetal radiation exposure, it is generally not recommended during pregnancy (54).

#### 3.2.2 Brain natriuretic peptide

BNP levels (>100 ng/L) correlate closely with adverse cardiac events, such as arrhythmias and endocarditis. BNP levels are influenced by multiple factors, including the timing of measurement, PAH severity, and cardiac function status in pregnant women. Regular monitoring facilitates the assessment of cardiac function; however, it must be integrated with other indicators for a comprehensive evaluation (48).

#### 3.2.3 New York heart association

NYHA class III–IV predicts adverse cardiac events; however, this classification is inherently subjective. Patients with moderate-to-severe PAH are more likely to present with corresponding symptoms, and their classification may be influenced by factors such as a history of arrhythmia. While regular monitoring can track changes in functional class, careful integration with other clinical variables is necessary (43).

#### 3.2.4 WHO functional classification assessment

The WHO functional classification is essential for evaluating exercise tolerance and cardiovascular disease severity in patients, including those with PAH. Monthly monitoring of symptoms and functional status is recommended during the first and second trimesters, with weekly assessments during the third trimester (55). Patients classified as functional class III–IV, denoting high risk, require enhanced supervisory measures.

#### 3.2.5 ESC/ERS 2022 risk assessment

This comprehensive risk stratification strategy, recommended in the ESC/ERS PAH guidelines, primarily serves to predict prognosis and inform treatment decisions. The model integrates multiple prognostic indicators, including WHO functional class, six-minute walking distance, BNP/NT-proBNP, and haemodynamic parameters, and stratifies patients into three-tier (low, intermediate, high) or four-tier (low, low-intermediate, intermediate-high, high) risk levels. For pregnant patients, this tool provides a reference framework for baseline risk assessment and longitudinal follow-up. During early and mid-gestation, a simplified assessment using non-invasive indicators (WHO functional class, six-minute walking distance, and BNP/NT-proBNP) can be performed every 4–6 weeks, enabling timely detection of escalating risk and facilitating adjustments to perinatal management. However, physiological haemodynamic changes and increased cardiac output during pregnancy may confound interpretation of some indicators, particularly BNP/NT-proBNP, while six-minute walking distance is affected by gestational age and weight gain. Therefore, assessment results should be interpreted in the context of individual clinical manifestations. Although large-scale validation studies in pregnant PAH patients are currently lacking, the stratification logic of this tool offers a quantifiable basis for clinical decision making (7).

The aforementioned ancillary assessment tools form a complementary hierarchy in clinical practice, spanning from initial screening to definitive diagnosis, and from baseline evaluation to dynamic monitoring. Echocardiography, as the preferred non-invasive initial screening and serial monitoring tool during pregnancy, can assess right heart structure and function; however, its estimation of pulmonary artery pressure carries a significant risk of bias, necessitating supplementation with biomarkers such as BNP and NT proBNP to provide corroborative evidence from the neurohormonal activation perspective (7). Although the NYHA/WHO functional class is relatively subjective, its simplicity and ease of use make it suitable as a basic assessment at each visit, complementing objective indicators. When non-invasive evaluation suggests disease progression or new clinical symptoms emerge, the ESC/ERS stratification framework can serve as an integrative tool for interpreting the aforementioned multidimensional indicators (functional class, biomarkers, hemodynamic parameters), helping to identify risk escalation and facilitate timely adjustment of management strategies. RHC, as the gold standard for diagnosis, although not recommended as a routine monitoring tool during pregnancy due to its invasiveness and radiation exposure, still holds irreplaceable value in specific situations such as uncertain diagnosis, difficulty in assessing treatment response, or preoperative final evaluation (56, 57). In summary, the rational selection and use of the aforementioned tools should be comprehensively decided by the MDT based on the patient’s disease evolution and gestational stage, in order to achieve a balance between precise assessment and minimisation of iatrogenic harm.

## 4 Multidisciplinary management strategy for pregnancies complicated by PAH

### 4.1 Composition and core responsibilities of the MDT

**Figure 1:**
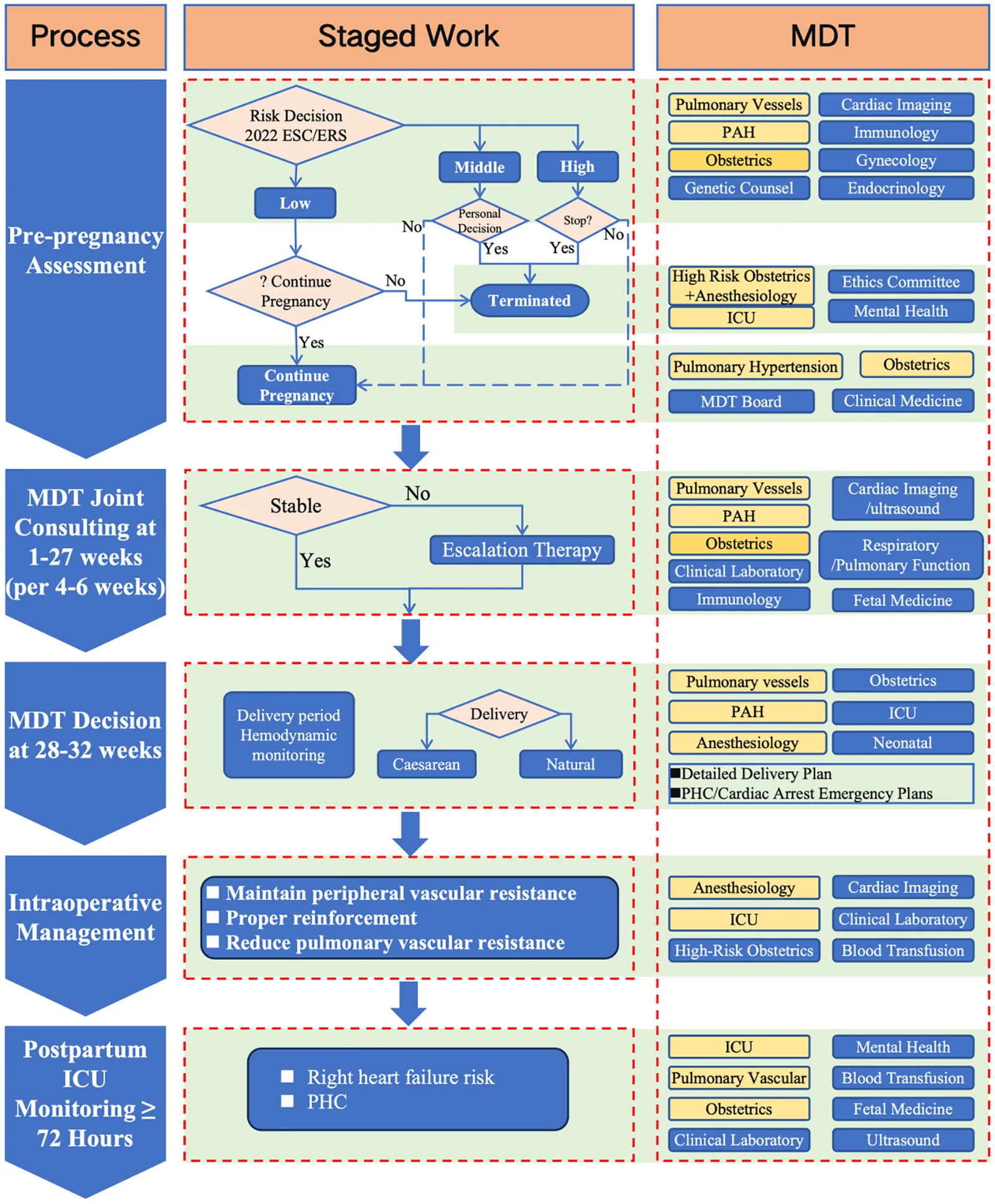
Schematic diagram of multidisciplinary team collaboration for the management of pregnancy complicated with pulmonary arterial hypertension.

**Figure 2:**
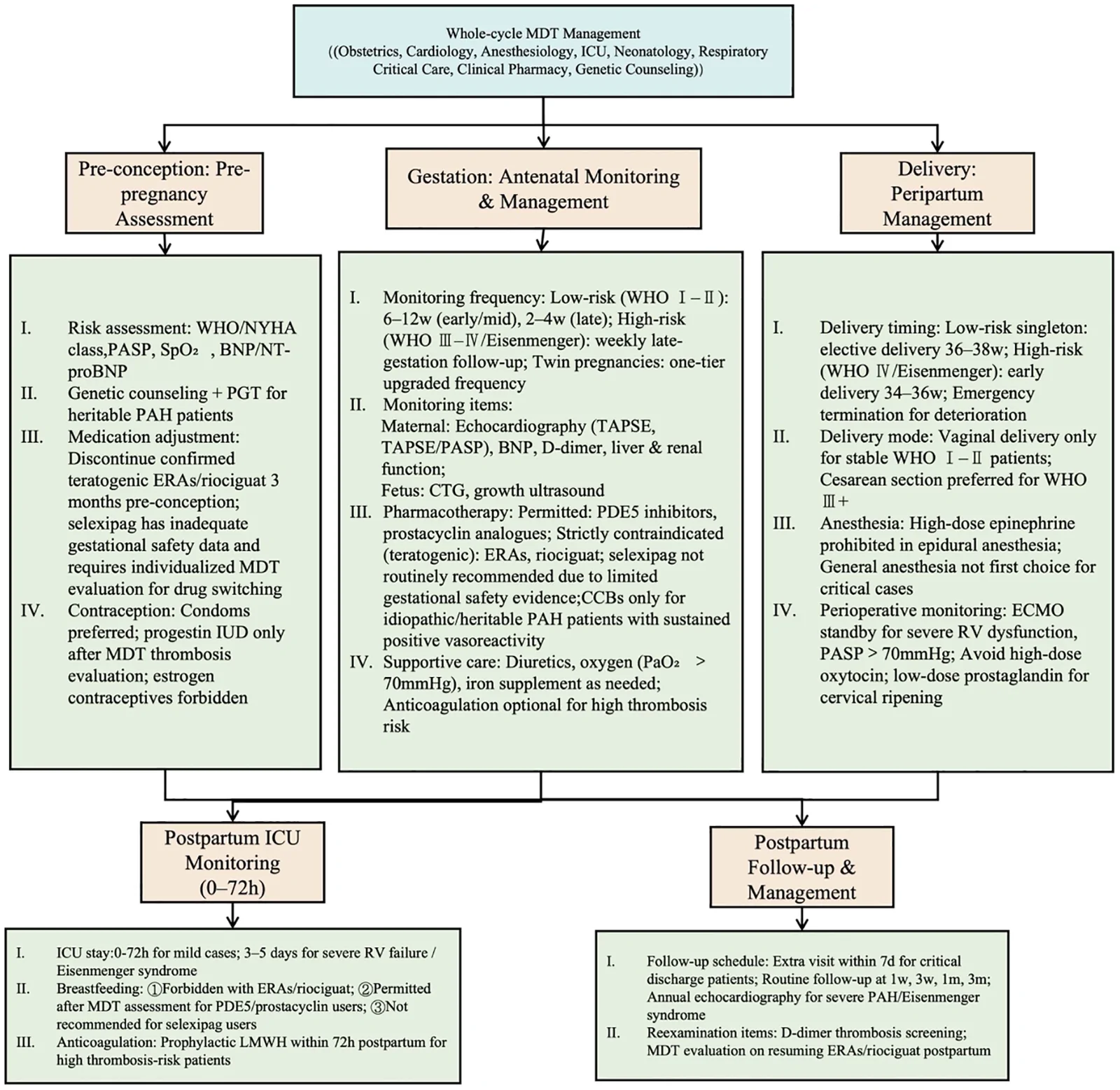
Whole-cycle multidisciplinary care pathway for pregnant patients with pulmonary arterial hypertension. BNP, B-type Natriuretic Peptide; CCBs, Calcium Channel Blockers; CTG, Cardiotocography; D-dimer, D-dimer; ECMO, Extracorporeal Membrane Oxygenation; ERAs, Endothelin Receptor Antagonists; ICU, Intensive Care Unit; LMWH, Low-Molecular-Weight Heparin; MDT, Multidisciplinary Team; NYHA, New York Heart Association; PAH, Pulmonary Arterial Hypertension; PASP, Pulmonary Artery Systolic Pressure,PDE5, Phosphodiesterase Type 5; PGT, Preimplantation Genetic Testing; PaO_2_, Partial Pressure of Oxygen; RV, Right Ventricular; TAPSE, Tricuspid Annular Plane Systolic Excursion; WHO, World Health Organization.

The MDT plays a crucial role in the comprehensive management of pregnancies complicated by PAH (48). Comprising experts from obstetrics, cardiology, anaesthesia, and neonatology, the team formulates personalised plans for prenatal care, delivery, and postpartum follow-up (58, 59) (**Figure 1**). The MDT’s primary responsibility is to weigh the maternal and foetal risks and benefits of continuing the pregnancy and to act promptly. Beyond this core group, the involvement of additional specialists is essential for optimising outcomes, particularly in high-risk patients. Intensivists with expertise in managing high-risk pregnancies are indispensable for perioperative and postpartum critical care, especially given that the majority of maternal deaths occur in the early postpartum period (5, 12). Pulmonologists or pulmonary hypertension specialists contribute to the optimisation of PAH-targeted therapies and long-term respiratory management (15). Clinical pharmacists play a vital role in medication reconciliation, ensuring the safe use of PAH-targeted drugs while avoiding teratogenic agents such as endothelin receptor antagonists and riociguat, and managing complex drug-drug interactions (14). Specialised nurses provide continuity of care, patient education, and coordination across multiple disciplines, which is particularly important given the chronic and complex nature of PAH (41). Furthermore, genetic counsellors should be involved when heritable PAH is suspected, as they facilitate pre-conception genetic counselling, interpret genetic test results, and guide reproductive decision-making, including preimplantation genetic testing (27, 45). This expanded multidisciplinary framework ensures that all aspects of maternal and foetal care—from genetic risk assessment and medication optimisation to critical care and long-term follow-up—are comprehensively addressed (**Figure 2**).

Three independent risk factors for postpartum PAH mortality have been identified: poor pre-pregnancy cardiac function, inadequate antenatal care, and hyperuricaemia (46). Accordingly, the MDT consultation must address the following elements: (a) management of severe cardiac events (e.g., pulmonary hypertensive crisis); (b) intraoperative invasive haemodynamic monitoring (pulmonary artery pressure, preload, central venous pressure); (c) anaesthesia selection; (d) postoperative analgesia; (e) targeted PAH and anticoagulant therapy; and (f) obstetric medication (60). A Chinese study demonstrated that MDT implementation reduced maternal mortality from 10.2% to 0%, with a 60% decrease in emergency caesarean sections and a 78% reduction in neonatal mortality, indicating substantially improved maternal and neonatal outcomes (61).

### 4.2 Pregnancy monitoring plan

Prenatal monitoring should adhere to the principles of stratification, dynamism, and multidisciplinary collaboration, implemented jointly by cardiology, obstetrics, anaesthesiology, and critical care medicine. The frequency and content of monitoring should be stratified according to the patient’s risk level. Each visit should include symptom assessment, physical examination, WHO/NYHA functional classification, weight and urine protein measurement, and foetal assessment (7).

#### 4.2.1 Low risk patients

During early and mid-pregnancy, patients require quarterly combined cardiology and obstetrics outpatient visits. In the third trimester (after 28 weeks of gestation), visits should be scheduled every 2–4 weeks. Echocardiography and BNP testing are recommended every 4–6 weeks (14).

#### 4.2.2 Moderate risk patients

Patients should attend the outpatient department every 2–4 weeks. NT-proBNP and blood oxygen saturation should be measured at intervals of two to four weeks. Echocardiography is recommended quarterly to assess RV function and pulmonary artery pressure. A six-minute walk test or cardiopulmonary exercise test should be performed for a comprehensive functional evaluation (14, 62).

#### 4.2.3 High/extremely-high risk patients

Hospitalisation or monitoring in a cardiac intensive care unit (CCU/ICU) with frequent monitoring (at least twice weekly) is recommended; daily monitoring may be required if necessary. These patients should undergo dynamic invasive or high-frequency non-invasive haemodynamic monitoring (e.g., cardiac output, central venous pressure), echocardiography every 1–2 weeks, and BNP/NT-proBNP, electrolyte, and liver/kidney function tests every 3–7 days. Vasoactive drugs should be readily available (14, 62).

#### 4.2.4 Monitoring critical gestational weeks and special circumstances

At 22 to 24 weeks of gestation, blood volume increases significantly; therefore, echocardiography and BNP assessment should be performed to facilitate timely treatment adjustments (17, 18). From 28 weeks onwards, foetal ultrasound assessments (including growth parameters, amniotic fluid volume, and umbilical artery blood flow) should be performed every 2–4 weeks. Between 32 and 34 weeks, blood volume peaks, representing the period of greatest maternal cardiac burden; hospitalisation or intensified monitoring in a day ward should be considered (17, 18). Before labour (34–36 weeks of gestation), a final pre-delivery assessment, comprising echocardiography, blood gas analysis, and coagulation function testing, should be conducted, along with a detailed delivery and anaesthesia plan (20). For high-risk patients, foetal heart rate monitoring and biophysical profile scoring are recommended. In cases of foetal distress or growth restriction, delivery should be expedited, and neonatal specialists should be involved in prenatal discussions (63).

Patients with hypoxaemia (SpO_2_<90%) require continuous oxygen therapy, in addition to daily blood gas and SpO_2_ monitoring, to rule out a right to left shunt or pulmonary embolism (49). Patients receiving diuretic or anticoagulant therapy should have electrolytes, renal function, and coagulation parameters (INR, PT, APTT) closely monitored (19). Patients with Eisenmenger syndrome require regular monitoring of arterial oxygen saturation and haematocrit, with particular attention to hyperviscosity syndrome and paradoxical embolism (22, 64).

The following warning signs should prompt immediate MDT discussion and consideration of early pregnancy termination or treatment escalation: (i) PASP increase of >15 mmHg from the previous measurement; (ii) BNP/NT-proBNP doubling within one week or persistently >200 ng/L; (iii) WHO functional class deterioration from I-II to III-IV; (iv) new-onset arrhythmia or syncope; (v) SpO_2_ decreasing to <90% despite oxygen therapy; and (vi) echocardiographic evidence of progressive RV enlargement or a significant reduction in TAPSE (34, 48, 53).

### 4.3 Pharmacotherapy

Pulmonary arterial hypertension can be alleviated through pharmacological intervention, particularly with targeted drugs. These agents fall into five distinct classes: endothelin receptor antagonists, phosphodiesterase type 5 (PDE5) inhibitors, calcium channel blockers, guanylate cyclase stimulators, and prostacyclin receptor agonists (65). According to the 2022 ESC/ERS PAH guidelines, prostaglandin analogues (e.g., epoprostenol, treprostinil, and iloprost), PDE5 inhibitors (e.g., sildenafil, vardenafil, and tadalafil), and calcium channel blockers (in patients with a positive acute vasoreactivity test) are considered relatively safe for use during pregnancy (7, 20). In contrast, endothelin receptor antagonists (e.g., ambrisentan, bosentan, and macitentan), guanylate cyclase stimulators (riociguat), and the prostacyclin receptor agonist selexipag are strictly contraindicated due to their teratogenic potential or unknown teratogenicity (7, 20). PDE5 inhibitors improve pulmonary vasodilation and inhibit vascular proliferation by enhancing nitric oxide-mediated vasorelaxation and elevating intracellular cGMP levels. Findings from limited small-sample trials and case reports constitute low-certainty evidence indicating that thrice-daily oral sildenafil (50–100 mg) might exert similar vasodilatory efficacy as inhaled nitric oxide in emergency PAH scenarios, though robust large-scale randomised controlled data are lacking (65).

The primary objective of prenatal PAH treatment is to preserve and optimise right ventricular function (42). Current stratified medication strategies are partially supported by formal clinical guidelines, while most individualised gestational dosing regimens are established on the basis of expert consensus and observational data, as large randomised controlled trials enrolling pregnant PAH patients remain lacking.

The primary objective of prenatal treatment is to optimise right heart function (42). The treatment plans that are implemented are subject to variation according to the severity of right heart dysfunction. Patients with preserved normal RV function: Oral PDE5 inhibitors (sildenafil, tadalafil) are recommended as first-line therapy, a uniform consensus proposal from PVRI’s expert panel (55). For patients who demonstrate a positive acute vasodilator test response, CCB maintenance can be added as an adjunct regimen, which is also a consensus-based management strategy without large prospective trial validation (66, 67). Mild RV dysfunction: Continuous oral or inhaled treprostinil is advised. This strategy lacks formal guideline endorsement and is only supported by accumulated retrospective observational data summarised in recent pregnancy-focused reviews (67). Severe RV dysfunction: Intravenous epoprostenol administered across gestation, labour and the postpartum period is proposed as rescue therapy. While this recommendation is noted in PVRI consensus, its supporting data solely consist of single-centre case cohorts and retrospective registries, with no high-quality prospective trial evidence to confirm its universal efficacy (55, 67).

### 4.4 Lifestyle and psychological support

The management of pregnancy in cases associated with PAH is a complex and detailed process. Whilst the importance of drug therapy cannot be overstated, it is imperative to recognise the significance of diet management, lifestyle adjustment, and psychological counselling (14).

PAH patients are predisposed to fluid retention and oedema, which exerts additional strain on the heart. Consequently, it is imperative to exercise stringent control over salt intake to avoid high-salt foods, such as pickled products, thereby mitigating water retention (55). To maintain electrolyte balance, it is necessary to increase dietary potassium intake appropriately. This can be achieved by consuming potassium-rich foods, such as bananas and spinach. In addition, it is essential to closely monitor the blood potassium level (14).

Bed rest is recommended to minimise unnecessary activity, thereby reducing cardiac burden, alleviating symptoms, and improving quality of life (55). The supine position should be avoided, as it allows the uterus to compress the inferior vena cava, reducing venous return and compromising circulation. The side-lying position is preferred to alleviate compression, enhance blood flow, and reduce cardiac workload (55).

PAH is a chronic disease whose symptoms have been observed to worsen during pregnancy. As indicated by the relevant literature, the physiological and psychological pressures experienced by pregnant women have been demonstrated to be a contributing factor to the onset of anxiety and depression (67). Consequently, the provision of timely and effective professional psychological counselling is imperative to maintaining their mental well-being. The provision of emotional support and understanding by family and social support networks is also invaluable in helping patients cope with the difficulties associated with pregnancy.

## 5 Delivery strategy and postpartum management

### 5.1 Selection of timing and method of childbirth

The primary delivery methods employed for pregnancies complicated by PAH are vaginal delivery and caesarean section. At present, a significant number of centres prefer caesarean section. A caesarean section has been shown to significantly shorten the duration of labour, with the potential to minimise haemodynamic fluctuations caused by prolonged uterine contractions and oxygen depletion. However, the decision regarding the timing and mode of delivery should be individualised based on a comprehensive assessment of both maternal and foetal factors.

From the maternal perspective, key determinants include right ventricular function and haemodynamic stability. Patients with severe right ventricular dysfunction, refractory heart failure, or progressive haemodynamic deterioration are generally considered poor candidates for vaginal delivery and may benefit from earlier, planned caesarean section (20, 55). Functional class is another important factor, as women with NYHA class III-IV symptoms, particularly those with worsening dyspnoea or signs of right heart failure, are at higher risk of intrapartum decompensation and often warrant caesarean delivery (68, 69). Pulmonary artery pressure level also plays a critical role, as patients with severe PAH (e.g., Pulmonary Artery Systolic Pressure ≥70 mmHg or mean pulmonary artery pressure ≥50 mmHg) may have a higher risk of peripartum complications, making a controlled caesarean delivery the preferred option (68). Foetal considerations include gestational age, foetal growth restriction, placental insufficiency, and foetal distress. Preterm delivery at 34–36 weeks is often considered to balance maternal cardiovascular risk and foetal maturity, particularly when the mother shows signs of haemodynamic compromise or when foetal well-being is concerning (20). However, if the maternal condition is stable and foetal surveillance is reassuring, continuation of pregnancy beyond 36 weeks with close monitoring may be considered on a case-by-case basis.

There is currently no consensus on the optimal timing of delivery, but most experts agree that a planned caesarean section at 34–36 weeks can balance maternal health and foetal maturity for high-risk patients (20). During this period, foetal organs are relatively mature, while the risk of maternal deterioration due to extended gestational age can be minimised. Nevertheless, vaginal delivery may be a reasonable option in carefully selected patients with favourable prognostic factors: those with mild-to-moderate PAH, preserved right ventricular function (e.g., tricuspid annular plane systolic excursion ≥16 mm, normal right atrial pressure), NYHA class I-II status, and reassuring foetal status (20, 68). In such cases, vaginal delivery is associated with lower risks of haemodynamic fluctuations, inflammatory stress, bleeding, infection, and air embolism compared with caesarean section (20, 68). For women who are not optimal candidates for vaginal delivery, a planned caesarean section under epidural or combined spinal-epidural anaesthesia, with continuous haemodynamic monitoring and availability of advanced therapies, remains the preferred approach (20, 55, 69).

### 5.2 Anaesthesia and haemodynamic management during childbirth

The selection of anaesthetic method has the capacity to exert a substantial influence on the prognosis of pregnant women with PAH. During childbirth, a variety of factors can influence fluctuations in pulmonary artery pressure. Such factors include the depth of anaesthesia, the method of anaesthesia, the mechanical ventilation settings, and hypoxaemia. When formulating the optimal anaesthesia strategy, factors such as pain control for the pregnant woman, haemodynamic stability, the oxygenation status of both the pregnant woman and the foetus, and the patient’s long-term prognosis must be considered comprehensively (6). In stable patients, epidural anaesthesia is commonly preferred and generally considered first-line, as it circumvents the myocardial decompensation that arises from a precipitous decline in systemic vascular resistance (70). However, the optimal choice may vary depending on individual patient characteristics and institutional practice. Conversely, the administration of general anaesthesia and positive-pressure ventilation has been shown to increase pulmonary vascular resistance and exacerbate right-to-left shunting in the heart. These risks are particularly pronounced in patients with severe PAH, right ventricular dysfunction, or significant intracardiac shunting, and are therefore condition-dependent rather than universally applicable. A recent study further suggests that the impact of general anaesthesia may vary depending on the presence of a cardiac shunt, being a significant predictor of adverse outcomes specifically in PAH patients without a shunt (71). Nevertheless, there are specific scenarios where general anaesthesia may be considered, such as in patients with contraindications to neuraxial blockade (9). However, it should be noted that the utilisation of general anaesthesia in pregnant women with congenital heart disease complicated by PAH has been associated with a substantially higher mortality risk, with some reports suggesting up to a fourfold increase (72).

The primary objective of treatment during childbirth is to maintain systemic and right atrial blood pressure, whilst monitoring fluid balance to avoid volume overload, particularly within the first 48 hours (55). In addition to right atrial pressure, more comprehensive endpoints such as cardiac output, systemic perfusion, and right ventricular function should be assessed to guide haemodynamic management (73). Postpartum, an elevation in right atrial pressure may signify excessive fluid accumulation within the body or worsening right ventricular function. The judicious utilisation of diuretics can facilitate effective management in such cases. In the event of patient deterioration, characterised by decreased central venous oxygen saturation and increased right atrial pressure, along with other indications of impaired pulmonary vascular function, escalation of intravenous prostaglandin therapy may be considered. Depending on the clinical phenotype and severity of right ventricular dysfunction, this can be administered concomitantly with positive inotropic drugs, such as low-dose dobutamine or milrinone in normotensive patients, for therapeutic purposes (74). In the event of symptoms suggestive of hypotension, a systematic investigation into potential causes is imperative. Such factors may include bleeding, sepsis, or heart failure resulting from low cardiac output or elevated afterload. Once the patient’s volume status has improved, Once the patient’s volume status has been optimised, vasopressor selection should be individualised: vasopressin or norepinephrine may be considered to improve the systemic-to-pulmonary vascular resistance ratio, with vasopressin offering the theoretical advantage of pulmonary vasodilatory effects in addition to systemic vasoconstriction (55, 74, 75).

For patients with severe PAH, such as those with Eisenmenger syndrome, supplemental treatments such as oxygen inhalation can be used during the perioperative period to reduce pulmonary artery resistance in patients who demonstrate vasoreactivity (55, 76). Concurrently, a target arterial oxygen tension of >70 mmHg has been suggested for pregnant women with PAH; however, this target should be interpreted with caution in conditions with right-to-left shunting (e.g., Eisenmenger syndrome), where baseline oxygen saturation is often lower and the relationship between PaO_2_ and clinical outcomes may differ (55, 64). Currently, there is insufficient research on the ideal haemoglobin level in this population.

### 5.3 Postpartum management

Pregnant women suffering from PAH must be closely monitored in the intensive care unit (ICU) in the days following delivery. Pregnant women frequently exhibit a hypercoagulable state, resulting in substantial fluid transfer and elevated catecholamine levels postpartum. Consequently, the body is often in a state of heightened compensatory response. Consequently, the majority of pregnancy-related fatalities transpire in the early postpartum period: a systematic review reported that approximately 61% of maternal deaths occurred within 0–4 days postpartum (5), while another multicentre retrospective study found that 78% of deaths occurred within the first month after delivery (72). The most prevalent etiologies encompass right heart failure and cardiovascular failure (5, 55). In patients with decompensated PAH, low-molecular-weight heparin may be considered for anticoagulation therapy to prevent thromboembolic events. Depending on clinical needs, concomitant administration of prostaglandin analogues (e.g., epoprostenol) may be used as an adjunct (67). A range of pharmacological agents may be used in the postpartum setting as clinically indicated, including: inotropes (e.g., dobutamine), vasopressors (e.g., norepinephrine, vasopressin), diuretics, sedative anaesthetic agents (e.g., propofol, etomidate, dexmedetomidine), PAH-targeted therapies (e.g., treprostinil in combination with sildenafil), and antibiotics (60). It should be emphasised that the use of inotropes and vasopressors should be individualised based on the patient’s haemodynamic phenotype and is generally reserved for cases with severe right ventricular dysfunction or systemic hypotension. During postpartum treatment, it is imperative to closely monitor maternal hemodynamic changes, including mean pulmonary artery pressure and mean arterial pressure, along with other crucial indicators, to avert sudden hemodynamic deterioration. It is recommended that patients receive regular follow-up care both during and after their hospitalisation, given that the impact of pregnancy on the cardiovascular system may persist for several months.

The decision regarding the initiation of breastfeeding postpartum should be made on an individualised basis under the guidance of the multidisciplinary team, taking into account the patient’s clinical circumstances, as drugs may be transferred to the infant through breast milk and theoretically carry potential risks. However, the available evidence is limited and derived predominantly from small case series. Although some studies have reported no significant adverse effects on the health status of breastfed infants whose mothers received sildenafil or bosentan (77, 78), such evidence is insufficient to establish long-term safety. Therefore, breastfeeding decisions should be individualised, and patients should be fully informed of the limitations of the current evidence.

## 6 Clinical management challenges and controversies

Pregnancy complicated by PAH is characterised by progressive pulmonary vascular lesions and specific haemodynamic changes during gestation. The entire diagnostic and therapeutic process spans the preconception, antenatal, delivery, and puerperium periods, and is constrained by multiple factors: physiological changes during pregnancy, teratogenic risks of medications, inconsistent diagnostic and treatment standards across centres, and disparities in primary care resources (57). Consequently, clinical practice continues to face numerous management challenges and unresolved academic controversies, which directly affect the implementation of risk stratification and multidisciplinary collaboration.

From an evidence-based perspective, the financial burden of PAH-targeted drugs is considerable; PAH medications are frequently costly and may be subject to additional requirements such as pregnancy testing before dispensing (79). The ongoing costs of treatment during pregnancy can be substantial—in China, the cost burden of iloprost alone has been estimated to account for over 8,000% of the disposable income of rural populations (80, 81). It has been observed that some patients independently reduce or discontinue their medication as a result of cost-related nonadherence, which may substantially increase the risk of heart failure and pulmonary hypertensive crisis; cost-related nonadherence has been associated with a 15%–22% increased risk of all-cause mortality in chronic disease populations (82). Furthermore, primary care security policies provide insufficient reimbursement coverage for PAH-targeted drugs during pregnancy, restricting standardised medication management throughout the entire process.

From a contextual and observational standpoint, most patients remain unaware of the fatal risks associated with PAH- complicated pregnancies. Low-risk patients may neglect regular cardiac function reviews, while high-risk patients may experience excessive anxiety and refuse necessary interventions.

Currently, psychological intervention is not yet routinely incorporated within the standard MDT management framework. This gap is concerning given the accumulating evidence that psychological factors are closely intertwined with clinical outcomes in this population. Cross-sectional studies have reported a high prevalence of anxiety (37%) and depression (28%) among PAH patients (83), and low positive affect has been independently associated with increased mortality (84). Experimental data also suggest a potential link between acute mental stress and elevated pulmonary vascular resistance, which may theoretically impose additional strain on the right ventricle (85). Nevertheless, these findings are predominantly associative, and whether psychological distress directly triggers pulmonary vasoconstriction-or merely reflects underlying disease severity-remains unclear. Future research integrating psychometric assessments with haemodynamic monitoring is needed to clarify these pathways and to determine whether structured psychological support could translate into measurable cardiovascular benefits in pregnant PAH patients.

Beyond the established pharmacological and MDT-based strategies, several emerging therapeutic avenues merit brief discussion, though their application in pregnancy-associated PAH remains largely experimental. Sotatercept, a first-in-class activin receptor type IIA-Fc fusion protein that reverses pulmonary vascular remodelling, has shown significant efficacy in the STELLAR trial (86)but is strictly contraindicated during pregnancy due to embryofetal toxicity observed in animal studies (FDA prescribing information for WINREVAIR^®^); women of childbearing potential must use effective contraception during treatment and for at least four months after the final dose. Mechanical circulatory support, including percutaneous right ventricular assist devices and veno-arterial extracorporeal membrane oxygenation (VA-ECMO), has been reported in isolated peripartum cases as salvage therapy for refractory right heart failure (87, 88), yet no prospective data guide optimal device selection or timing in this population. Gene therapy and nucleic acid-based approaches targeting BMPR2 mutations remain preclinical, but their potential, combined with preimplantation genetic testing, may one day inform reproductive decision-making for heritable PAH (27). Digital health tools, such as wearable devices for continuous SpO_2_and heart rate monitoring, offer promise for remote surveillance of high-risk pregnant patients (89), though validation specifically in gestation is lacking. Collectively, these innovative approaches underscore the urgent need for international, prospective pregnancy registries to systematically capture maternal and foetal outcomes, enabling evidence-based integration of emerging therapies into clinical practice (14).

## 7 Summary and key points of clinical practice

Pregnancy in women with PAH carries significant maternal health risks. The core principles of management can be summarised as follows, with the understanding that specific practices should be adapted to local resources and multidisciplinary team expertise:

### 7.1 Preconception and early pregnancy

Comprehensive counselling is recommended for all women of childbearing age with PAH, covering disease aetiology, haemodynamic status, and maternal-foetal risks. MDT involvement before conception is advised to develop an individualised plan. Risk stratification should include PAH aetiology, PASP, WHO functional class, BNP/NT-proBNP, and SpO_2_.

### 7.2 Antenatal monitoring

Multidisciplinary follow up is advised throughout pregnancy, with low risk patients seen every 4–6 weeks in combined clinics and intermediate/high risk patients receiving intensified surveillance (inpatient or ICU) based on clinical status and institutional protocols. Monitoring frequency should be dynamically adjusted according to functional class, biomarker trends, and haemodynamic changes.

### 7.3 Delivery strategy

Delivery timing and mode should be individualised based on maternal cardiac status, obstetric factors, and foetal maturity. Elective delivery at 34–36 weeks is commonly chosen to avoid late-gestation decompensation, though the final decision rests with the MDT. Neuraxial anaesthesia is preferred for its hemodynamic stability, but the final plan should be tailored to the patient and institutional expertise.

### 7.4 Postpartum care

Continuous monitoring in a critical care setting for ≥72 hours is recommended, as most maternal deaths occur in this period. Focus on right ventricular function, fluid balance, and thromboprophylaxis, with early treatment escalation if deterioration occurs.

### 7.5 Comorbidity management

For secondary PAH, collaborative management with relevant specialties (e.g., rheumatology, hepatology, infectious diseases) is advised to optimise underlying disease control, but pregnancy management should remain guided by PAH severity stratification.

### 7.6 Patient education

All women of childbearing potential with PAH should receive genetic counselling (when indicated) and contraception education to prevent unplanned pregnancies, ideally as part of routine long-term care in PAH specialty centres.

## Glossary

APTT: Activated Partial Thromboplastin Time
BNP: Brain Natriuretic Peptide
CCB: Calcium Channel Blocker
CCU: Cardiac Care Unit
cGMP: cyclic Guanosine Monophosphate
CHD: Congenital Heart Disease
CTD: Connective Tissue Disease
CTEPH: Chronic Thromboembolic Pulmonary Hypertension
CTG: Cardiotocography
ERA: Endothelin Receptor Antagonist
ERS: European Respiratory Society
ESC: European Society of Cardiology
HHT: Hereditary Hemorrhagic Telangiectasia
ICU: Intensive Care Unit
INR: International Normalised Ratio
iPAH: idiopathic Pulmonary Arterial Hypertension
IVF: *In Vitro* Fertilisation
LMWH: Low-Molecular-Weight Heparin
MACE: Major Adverse Cardiovascular Events
MDT: Multidisciplinary Team
mWHO: modified World Health Organization
NICU: Neonatal Intensive Care Unit
NT-proBNP: amino-N-terminal Pro-brain Natriuretic Peptide
NYHA: New York Heart Association
PaO₂: Partial Pressure of Oxygen, arterial
PAH: Pulmonary Arterial Hypertension
PASP: Pulmonary Artery Systolic Pressure
PDE5: Phosphodiesterase Type 5
PGT: Preimplantation Genetic Testing
PGT-M: Preimplantation Genetic Testing for Monogenic Disorders
PHC: Pulmonary Hypertensive Crisis
PT: Prothrombin Time
PVR: Pulmonary Vascular Resistance
PVRI: Pulmonary Vascular Research Institute
RHC: Right Heart Catheterisation
RHF: Right Heart Failure
RV: Right Ventricular
SLE: Systemic Lupus Erythematosus
SpO₂: Peripheral Oxygen Saturation
SSc: Systemic Sclerosis
TAPSE: Tricuspid Annular Plane Systolic Excursion
VA-ECMO: Veno-Arterial Extracorporeal Membrane Oxygenation
WHO: World Health Organization
